# The Role of Therapeutic Leukapheresis in Hyperleukocytotic AML

**DOI:** 10.1371/journal.pone.0095062

**Published:** 2014-04-14

**Authors:** Friederike Pastore, Alessandro Pastore, Georg Wittmann, Wolfgang Hiddemann, Karsten Spiekermann

**Affiliations:** 1 Department of Internal Medicine III, University Hospital Munich, Ludwig-Maximilians-University Munich - Campus Groβhadern, Munich, Germany; 2 Department of Transfusion Medicine, University Hospital Munich, Ludwig-Maximilians-University Munich - Campus Groβhadern, Munich, Germany; 3 German Cancer Consortium (DKTK), Heidelberg, Germany; 4 German Cancer Research Center (DKFZ), Heidelberg, Germany; University of Colorado School of Medicine, United States of America

## Abstract

**Purpose:**

Hyperleukocytosis in AML with leukostasis is a serious life-threatening condition leading to a high early mortality which requires immediate cytoreductive therapy. Therapeutic leukapheresis is currently recommended by the American Society of Apheresis in patients with a WBC>100 G/l with signs of leukostasis, but the role of prophylactic leukapheresis before clinical signs of leukostasis occur is unclear.

**Patients:**

We retrospectively analyzed the role of leukapheresis in 52 patients (median age 60 years) with hyperleukocytotic AML with and without clinical signs of leukostasis. Since leukapheresis was performed more frequently in patients with signs of leukostasis due to the therapeutic policy in our hospital, we developed a risk score for early death within seven days after start of therapy (ED_d7_) to account for this selection bias and to independently measure the effect of leukapheresis on ED_d7_.

**Results:**

20 patients received leukapheresis in combination to chemotherapy compared to 32 patients who received chemotherapy only. In a multivariate logistic regression model for the estimation of the probability of ED_d7_ thromboplastin time and creatinine remained as independent significant parameters and were combined to create an ED_d7_ risk score. The effect of leukapheresis on EDd7 was evaluated in a bivariate logistic regression together with the risk score. Leukapheresis did not significantly change early mortality in all patients with a WBC≥100 G/l.

**Discussion:**

Prophylactic leukapheresis in hyperleukocytotic patients with and without leukostasis did not improve early mortality in our retrospective study. Larger and prospective clinical trials are needed to validate the risk score and to further explore the role of leukapheresis in AML with hyperleukocytosis.

## Introduction

Hyperleukocytosis is defined as white blood count (WBC)≥50–100 G/l and occurs in 5–18% of AML [1]. Hyperleukocytosis is often associated with the FAB subtypes M4/M5 and has been shown to be a risk factor for an adverse outcome, a high early death (ED) rate [2] and high relapse rate.

Critical hyperleukocytosis can cause leukostasis, a life-threatening condition with disturbance of microcirculation caused by occlusion of small vessels due to elevated WBC, endothelial adhesion of myeloid blasts and tissue infiltration [3].

Besides the high blood viscosity as a physical mechanism for the microcirculatory disturbances, blast cells have been shown to secrete cytokines (IL-1β and TNF-β) leading to an up-regulation of endothelial adhesion molecules (ICAM-1, VCAM-1, E-selectin) and thus a concomitant adhesion of blasts on the endothelium [3]. Elevated serum lactate levels might represent an early sign of microcirculatory failure [4].

Clinically, leukostasis syndrome most commonly affects the lungs in about 80%, the central nervous system and the kidneys in a rapid progressive often fatal course of disease [5], [6]. As treatment of choice, a rapid reduction of the WBC is thought to be mandatory. Therefore chemotherapy is often combined with therapeutic leukapheresis, a physical method to reduce high WBC and blood viscosity.

Leukapheresis is known to be a generally safe procedure. Nevertheless, the placement of a large central venous catheters required for leukapheresis might bear an elevated risk of bleeding, especially since patients are often thrombocytopenic and suffer from coagulopathy [7].

Most authors agree, that leukapheresis does not seem to have an impact on long-term outcome in hyperleukocytotic AML patients with leukostasis [1], [8], [9]. Nevertheless, the role of leukapheresis on early mortality is contradictory in different studies [1], [7]–[10] and has not been investigated in prospective clinical studies yet.

The aim of our study was to investigate the role of leukapheresis in hyperleukocytotic AML patients treated in our clinical center.

## Materials and Methods

### Patients

Between January 1999 and August 2012 we identified 69 previously treated or untreated AML patients older than 18 years with hyperleukocytosis (WBC≥100 G/l) admitted to our hospital by a laboratory screen (clinical chemistry data bank). Identified patients were cross-checked with medical records, discharge letters, diagnostics performed in our leukemia laboratory and the leukapheresis records in the department of transfusion medicine. Diagnoses included de novo AML, secondary and therapy-related AML.

Based on the number of 102 AML patients treated in our hospital from Januar 2012 until December 2013 and documented in the AML registry, we have treated about 727 AML patients in the period from January 1999 until August 2012.

Thus, the estimated rate of hyperleukocytotic AML in our center was 9.5%, which is in the range of published data.

The study was approved by our local institution (Internal medicine III, University Hospital LMU Munich). According to the European legislation no written consents are necessary for observational retrospective studies if data contain no personal identifiers and data are analyzed anonymously. Therefore, and because of the retrospective study design, informed consent was waived. All clinical investigations were conducted in accordance with the guidelines of the 2008 Declaration of Helsinki.

### Diagnosis

AML diagnosis was established on the basis of standard morphologic and cytochemical examinations of peripheral blood and marrow smears according to the French-American-British (FAB) and the World Health Organization (WHO) criteria.

Furthermore cytogenetic analysis [11], FISH, immunophenotypic analysis of marrow aspirates was performed. Screening of established molecular markers e.g. mutations of *NPM1*, *FLT3-ITD*, *FLT3*-TKD and *MLL-PTD* was performed according standard protocols [12]–[17].

### Statistics

Baseline comparisons between patients with and without therapeutic leukapheresis were calculated using the Χ^2^ for dichotomous variables and the Mann Whitney U test for continuous parameters with SPSS version 20.0. Outcome parameters were calculated using logistic regression and Kaplan Meier plots. Median follow up was calculated using the reversed Kaplan Meier method.

For the assessment of the prognostic effect on ED each parameter was introduced in a univariate logistic regression. All significant univariate parameters (p-value <0.05) where tested pairwise in a bivariate logistic regression model. All significant parameters in bivariate analyses were introduced into a multivariate logistic regression model (p-value <0.05 required for inclusion in multivariate model). Multivariate logistic regression was performed with inclusion of all significant parameters without stepwise selection [18]. To account for the bias of small numbers, a bootstrap multivariate logistic regression model with 999 bootstrap replications was performed [19]. The covariates that remained independently significant in both multivariate models with a significance level of 5%, were used for the establishment of an ED risk score. Their regression coefficients were used for the calculation of the early mortality risk score [18]. For the estimation of the diagnostic quality of the risk score in terms of prediction of ED with a very high specificity and sensitivity and for the determination of the cutoff a receiver operating characteristic (ROC) curve analysis was performed. This area under the curve (AUC) illustrates the test performance reaching from 0.5 ( =  of no diagnostic value) to 1.0 ( =  best diagnostic value). For identification of the optimal cutoff between a low risk and a high risk group we defined to achieve a sensitivity and specificity of >75%.

### Therapy

All intensively treated patients started with a chemotherapy with low dose cytarabine (100 mg/m^2^ or 100 mg absolute) at the day of admission.

Depleting leukapheresis as initial therapeutic regime in hyperleukocytotic AML patients was performed additionally to chemotherapy in patients with laboratory signs of leukostasis e.g. elevated lactate level or elevated troponin since 2004. Between 1999 and 2003 institutional policy for the treatment of hyperleukocytosis did not involve leukapheresis as a routine treatment.

### Endpoints

Primary endpoints of our retrospective investigation was mortality within 7 days, after 4 weeks and overall survival (OS), after diagnosis of hyperleukocytosis. Death within the first 7 days was defined as “early death” (ED_d7_). Secondary endpoints were event-free survival (EFS) for all patients and cumulative incidence of relapse (CIR) for all patients in complete remission (CR). In patients who had undergone allogeneic transplantation OS, EFS and CIR were censored at the start of transplantation.

Furthermore we investigated the degree of blast clearance measured in the bone marrow one week after first induction treatment and the achievement of a complete remission.

## Results

### Patient characteristics

**Figure 1:**
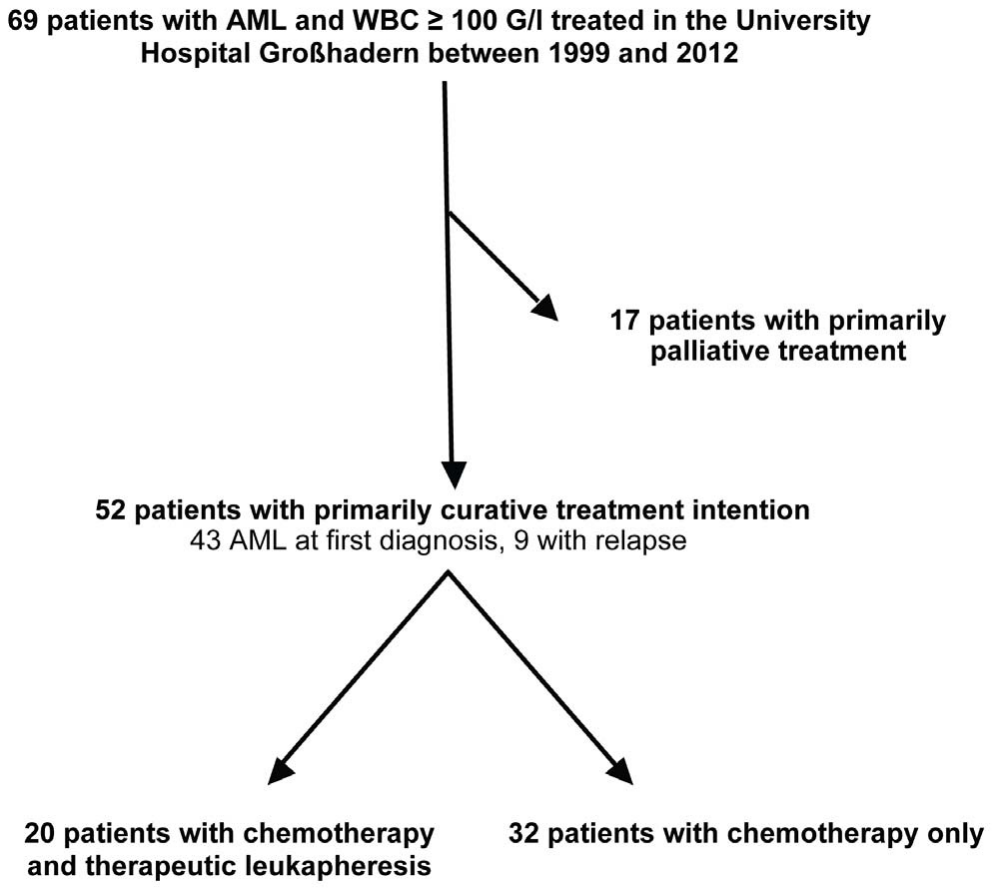
Overview of patient selection.

Fifty-two of 69 patients underwent intensive therapy in a curative intention. In the majority of patients (n = 43/52; 83%) hyperleukocytosis was present at first AML diagnosis. In 9 patients hyperleukocytosis occured at the time of relapse. Twenty patients received chemotherapy and therapeutic leukapheresis, whereas 32 patients were treated with chemotherapy alone (**Figure 1**).

Seventeen patients who underwent primarily palliative treatment where excluded from further analyses. Reasons for palliative treatment at the time of first AML diagnosis included comorbidities (second advanced metastatic cancers e.g. coecum cancer, rectal cancer; severe heart insufficiency NYHA >III, terminal chronic renal failure), age >85 years and reduced baseline performance status according to the Eastern Cooperative Group (ECOG 4).

81% of the 52 intensively treated patients had de novo AML. Secondary AML arising from a myelodysplastic or myeloproliferative syndrome was diagnosed in 6% and therapy-related AML due to a chemotherapy or radio- therapy for a previous malignancy in 13% of patients.

Median age was 60 years, 58% were female. The majority of patients had an Eastern Cooperative Group (ECOG) performance status of 3 or 4 when admitted to the hospital (**Table 1**). Median WBC was 159 G/l and median bone marrow blasts 87%. 45% had an AML M4 or M5 according to the FAB classification of AML.

**Table 1:** Patient characteristics of all intensively treated patients with WBC≥100 G/l.

		n = 52	
characteristic	n		%
**Age (years)**			
** median**		**60**	
** range**		**21–79**	
**Female sex**	**30**		**58**
**ECOG 3/4**	**37**		**84**
**De novo AML**	**42**		**81**
**First diagnosis**	**43**		**83**
**WBC (G/l)**			
** median**		**159**	
** range**		**100–322**	
**Platelets (G/l)**			
** median**		**45**	
** range**		**10–149**	
**Hemoglobin level (g/dl)**			
** median**		**9.5**	
** range**		**5.0–14.9**	
**LDH level (U/l) n = 46**			
** median**		**996**	
** range**		**444–7387**	
**BM blasts (%) n = 40**			
** median**		**87**	
** range**		**46–99**	
**PB blasts (%) n = 47**			
** median**		**84**	
** range**		**14** * **–99**	

Patients with hyperleukocytosis had a reduced ECOG performance status and highly elevated WBC, LDH level and blast counts.

Abbreviations: BM blasts; bone marrow blasts; EOCG, Eastern Cooperative Group; LDH, lactase dehydrogenase; PB blasts; blasts in the peripheral blood; WBC, white blood count.

*one patient with an AML M5A showed 14% of myeloblasts in the peripheral blood, not accounting for the 59% of monoblasts in AML M5.

Pretreatment cytogenetic results were available for 49 patients (94%). By use of the cytogenetic classification of the ECOG [11] 4% had favorable, 84% had intermediate, and 12% had adverse-risk karyotypic abnormalities. According to the ELN classification, 66% of hyperleukocytotic patients belonged to the favorable and Intermediate-I-risk group [20]. 61% were cytogenetically normal. An overview of morphologic, cytogenetic and molecular abnormalities is provided in **Table 2** **.** Mutational analyses of both, *NPM1* and *FLT3-ITD*, were available in 79% of patients.

**Table 2:** Morphologic, cytogenetic and molecular characteristics of patients with WBC≥100 G/l.

characteristic	n	%
FAB M4/M5	23/51	45
**Cytogenetics**		
** Cytogenetically normal**	**31/49**	**63**
** ** ***PML-RARA***	**2/49**	**4**
** CBF-AML**	**2/49**	**4**
** ** ***MLL*** **-rearrangements**	**5/49**	**10**
** Complex karyotype**	**4/49**	**8**
** other**	**5/49**	**10**
**ELN risk**		
** ELN favorable**	**10/45**	**22**
** ELN intermediate-I**	**20/45**	**44**
** ELN intermediate-II**	**9/45**	**20**
** ELN adverse**	**6/45**	**13**
**Molecular genetics**		
** ** ***NPM*** **1+**	**19/41**	**46**
** ** ***FLT3*** **-ITD+**	**20/46**	**44**
** ** ***FLT*** **3-TKD+**	**3/38**	**8**
** ** ***MLL*** **-PTD+**	**2/42**	**5**

Hyperleukocytotic patients commonly had AML FAB types M4/M5 or ELN favorable or intermediate-I risk.

Abbreviations: FAB, French-American-British classification of AML; ELN, European Leukemia Net classification of AML; *FLT*3-ITD, internal tandem duplication of the *FLT*3 gene; *FLT*3-TKD, point mutation at D835 in the *FLT*3-tyrosine kinase domain of the *FLT*3 gene; *MLL*-PTD, partial tandem duplication of the *MLL* gene; n, number; *NPM*1, nucleophosmin1.

More than one third of patients showed clinical signs of leukostasis, such as dyspnea (44%), acute renal failure (25%) and neurologic symptoms (8%). Troponin and lactate levels were elevated in 65% and 23% of patients respectively (**Table 3**).

**Table 3:** Clinical manifestations of hyperleukocytosis/leukostasis in all patients, patients with therapeutic leukapheresis plus chemotherapy and patients with chemotherapy only.

		All (n = 52)			LA/Chemo (n = 20)			Chemo (n = 32)		*P* LA/Chemo vs Chemo
characteristic	n		%	n		%	n		%	
**Creatinine (mg/dl)**										
** median**		**1.2**			**1.2**			**1.1**		**n.s.**
** range**		**0.6–3.5**			**0.6–3.0**			**0.6–3.5**		
**Troponin (ng/ml) n = 23**										
** median**		**0.06**			**0.12**			**0.05**		**0.039**
** range**		**0.05–10.90**			**<0.05–10.90**			**<0.05–3.99**		
**Troponin (>0.05 ng/ml) n = 23**	**15**		**65**	**12/14**		**86**	**3/9**		**33**	**0.010**
**Lactate (mmol/l) n = 31**										
** median**		**1.4**			**1.7**			**1.2**		**n.s.**
** range**		**0.3–36.2**			**0.3–36.2**			**0.6–4.2**		
**Lactate (>2.4 mmol/l) n = 31**	**7**		**23**	**4/13**		**31**	**3/18**		**17**	**n.s.**
**PTT (sec) n = 51**										
** median**		**35**			**33**			**36**		**n.s.**
** range**		**20–141**			**20–141**			**24–59**		
**Thromboplastin time (%)**										
** median**		**60**			**51**			**63**		**0.005**
** range**		**6–85**			**6–80**			**39–85**		
**Fibrinogen (mg/dl) n = 35**										
** median**		**253**			**190**			**289**		**n.s.**
** range**		**52–577**			**52–484**			**71–577**		
**Antithrombin (%) n = 31**										
** median**		**89**			**73**			**95**		**0.002**
** range**		**11–129**			**11–113**			**58–129**		
**D-dimer (µg/ml) n = 15**										
** median**		**26.9**			**17.9**			**32.1**		**n.s.**
** range**		**0.0–40.0**			**1.7–40.0**			**0.0–40.0**		
**Dyspnea n = 46**	**20**		**44**	**14/20**		**70**	**6/26**		**23**	**0.001**
**Oxygen requirement n = 46**										**0.008**
** none**	**26**		**57**	**6/20**		**30**	**20/26**		**77**	
**nasal prongs (≤4l O2/min)**	**10**		**22**	**7/20**		**35**	**3/26**		**12**	
**oxygen mask (>5l O2/min)**	**7**		**15**	**4/20**		**20**	**3/26**		**12**	
** CPAP/ITN**	**3**		**6**	**3/20**		**15**	**0/26**		**0**	
**Neurologic derogation n = 50**	**4**		**8**	**4/20**		**20**	**0/30**		**0**	**0.011**
**Acute renal failure/HD/HF/Creatinine >1.5 mg/dl**	**13**		**25**	**7**		**35**	**6**		**19**	**n.s.**

Patients receiving leukapheresis and chemotherapy showed significantly more clinical symptoms of leucostasis and a deregulated coagulation compared to the patients receiving chemotherapy only. This reflects the policy of leukapheresis in our hospital, since only patients with signs of leukocytosis underwent leukapheresis.

Abbreviations: CPAP, continuous positive airway pressure; HD, hemodialysis; HF, hemofiltration; ITN, intubaton; LA, leukapheresis; PTT, partial thromboplastin time.

All patients started with a chemotherapy at the day of hospital admission. 23% received only cytarabine prephase (100 mg/m^2^ or 100 mg absolute) since they died before the start of the first induction course, 33% received one induction course and 44% received double induction chemotherapy (**Table S1**). 33% of patients received allogeneic stem cell transplantation. 29% of patients were treated within clinical studies (AMLCG99 [NCT00266136] n = 5, AMLCG2004 [21] n = 4, AMLCG2008 [NCT01382147] n = 7).

Additional therapeutic leukapheresis procedures were performed in 20 of 52 patients. In 18/20 patients leukapheresis was performed once. One patient underwent two and one patient underwent three leukapheresis on a daily basis. In 18/20 patients (90%) therapeutic leukapheresis was performed at day 0 or 1 after admission to the hospital, 2 patients underwent leukapheresis on day 2 and 4 respectively.

32 of 52 patients with hyperleukocytosis received chemotherapy without leukapheresis.

**Figure 2:**
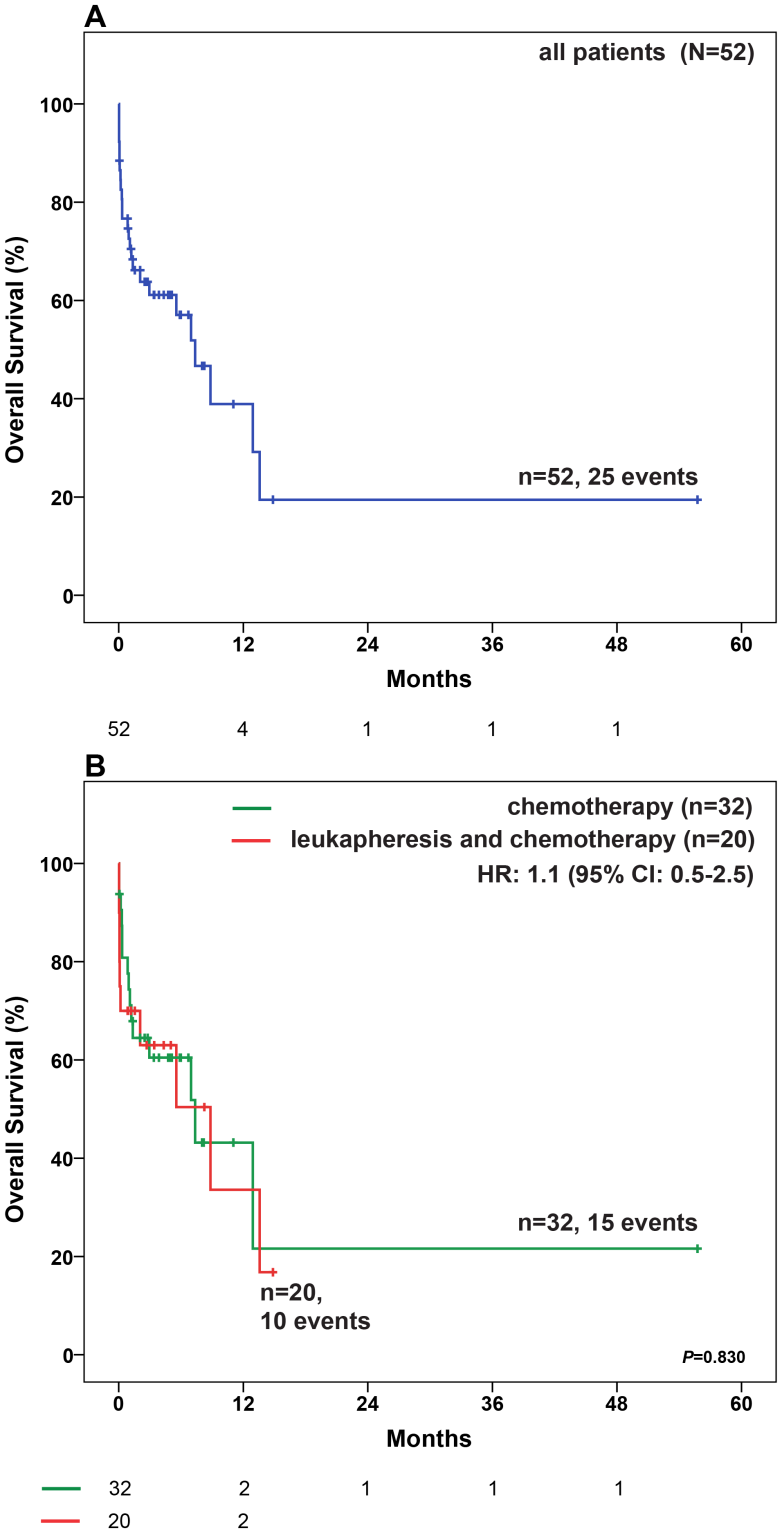
OS in patients with WBC≥100 G/l. (A) in all patients (B) in patients who received either chemotherapy only or chemotherapy combined with leukapheresis. Median OS was 7.4 months (95% CI: 4.1–10.6 months) in all patients, 7.4 months (95% CI: 1.3–13.4 months) in patients receiving chemotherapy only and 8.8 months (95% CI: 1.3–16.4 months) in patients with the combination of chemotherapy and leukapheresis. Abbreviations: CI, confidence interval; HR, Hazard Ratio, OS, Overall survival;

Median follow up for all patients was 14.9 months (95% confidence interval (CI): 6.3–23.4 months). Median overall survival (OS) was 8.8 months (95% CI: 5.4–12.3 months) in all patients uncensored for allogeneic transplantation and 7.4 months (95% CI: 4.1–10.6 months) when patients were censored at the time of allogeneic transplantation (**Figure 2A**). 8% of patients died within the first 24 hours and 17% within the first seven days after the diagnosis of hyperleukocytosis.

### Comparison between patients with and without therapeutic leukapheresis

Since the policy in our hospital was to perform leukapheresis in patients with signs of leukocytosis, there were significant differences with regard to leukostasis parameters such as elevated troponin, dyspnea, oxygen need, neurologic disturbances at initial presentation between the 20 patients that were treated with leukapheresis and chemotherapy and the 32 patients with chemotherapy only (**Table 3**). Furthermore WBC (208 G/l vs. 142 G/l, p = 0.023) and LDH levels (1568 U/l vs. 845 U/l, p = 0.030) were significantly higher in the patients with leukapheresis illustrating the rapid turnover of immature cells. Coagulation was significantly disturbed in the leukapheresis group demonstrated by lower prothrombin time and antithrombin levels (**Table 3**).

There were no statistically significant differences in patients' performance status, sex or the type of AML (de novo AML, AML at first diagnosis, FAB type, cytogenetic risk group, ELN risk group or molecular characteristics). Also, median age in patients receiving leukapheresis combined with chemotherapy (62 years, range 21–79 years) and those receiving chemotherapy only (60 years, range 31–79 years), was not significant different (p = 0.351).

The number of induction cycles was significantly different between the two cohorts, especially in patients who did not receive a full induction cycle due to death or life threatening complications (40% in the leukapheresis group vs. 9% in the chemotherapy group).

**Figure 3:**
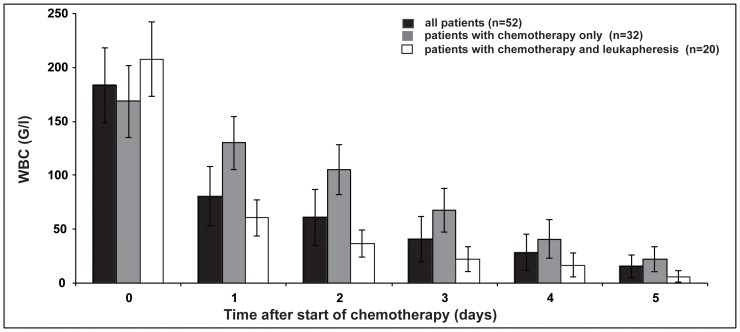
Efficacy of reduction of WBC with chemotherapy only and chemotherapy combined with leukapheresis. Abbreviations: WBC, white blood count.

Leukapheresis was a potent procedure to efficiently reduce WBC below 50 G/l significantly faster compared to chemotherapy alone (median 1 day [0–5 days] compared to 4 days [2–14 days] p<0.001) (**Figure S1,** **Figure 3**). Median reduction of the WBC after one leukapheresis was 77% (42%–90%).

Although patients that underwent therapeutic leukapheresis showed a significant faster reduction of WBC, they displayed a higher death rate within the first 24 hours and a trend to a higher early death rate compared to patients with chemotherapy alone (**Table 4**).

**Table 4:** Outcome in all patients, patients with therapeutic leukapheresis plus chemotherapy and patients with chemotherapy only.

		All (n = 52)			LA/Chemo (n = 20)			Chemo (n = 32)		P LA/Chemo vs Chemo
endpoint	n		%	n		%	n		%	
**Death**										
**≤24 hours**	**5**		**10**	**4**		**20**	**1**		**3**	**0.045**
** ≤7 days**	**9**		**17**	**6**		**30**	**3**		**9**	**0.056**
** ≤28 days**	**13**		**25**	**6**		**30**	**7**		**22**	**n.s.**
** ≤100 days**	**19**		**37**	**7**		**35**	**12**		**38**	**n.s**
** ≤ last follow up**	**25**		**48**	**10**		**50**	**15**		**47**	**n.s.**
**Adequate early blast clearance (n = 45)**	**28**		**62**	**9**		**56**	**19**		**66**	**n.s.**
**Complete remission (n = 47)**	**25**		**53**	**8**		**50**	**17**		**55**	**n.s.**
**Relapse (n = 25)**	**13**		**52**	**4**		**50**	**9**		**53**	**n.s.**

Patients that were treated with leukapheresis and chemotherapy had a higher death rate in the first 24 hours and first 7 days compared to patients receiving chemotherapy only. Reasons for lack of evaluation for remission status in 5 patients were a) no diagnostic bone marrow biopsy due to patient desire (n = 1) b) loss of follow-up (n = 1), end of follow- up (n = 1), no complete induction therapy (n = 2).

Abbreviations: LA, leukapheresis, n, number.

Achievement of an adequate early blast cell clearance or a complete remission as well as long term outcome (OS, EFS, CIR) were not different between the two cohorts (**Figure 2B** **, Figures S2 and S3**).

In both cohorts, older age was not a significant risk factor for a higher ED_d7_ rate or an impaired OS (data not shown).

### Early Complications

Severe complications in the first seven days after the start of the treatment were mainly bleeding events or thromboembolic complications (**Table 5**).

**Table 5:** Complications within 7 days of treatment.

Complication		LA/Chemo (n = 20)			Chemo (n = 32)		*P*
	n		%	n		%	
**Bleeding**	**7**		**35**	**5**		**16**	**n.s.**
** Cerebral bleeding**	**3**		**15**	**2**		**6**	**n.s.**
** Life threatening bleeding**	**2**		**10**	**2**		**6**	**n.s.**
**Thrombosis/Embolism**	**3**		**15**	**0**		**0**	**0.024**
** Without splenic infarction** *	**2**		**10**	**0**		**0**	**n.s.**

There was a trend to more bleeding events and thromboembolic events within the patients receiving leukapheresis and chemotherapy.

*exact time point of the first manifestation splenic infarction is not clear; it was diagnosed in a CT scan performed after leukapheresis.

Abbreviations: LA, leukapheresis.

Of all 52 patients, 5 experienced an intracerebral bleeding. Other major causes of bleeding events were located in the upper gastrointestinal tract (n = 1), bleeding in the pericard (n = 1), the pleura (n = 1) and the ascites (n = 1), makrohematuria (n = 1) and bleeding at central venous catheter puncture sites (n = 2). There was no statistical difference in the frequency of bleeding events between patients with and without leukapheresis.

Three patients developed thromboembolic complications such as pulmonary embolism, ischemic cerebral infarction as complication of a total occlusion of the carotid artery and splenic infarction.

In the two patients with the pulmonary embolism and the artery occlusion, the event occurred within 24 hours after leukapheresis.

The frequency of major thromboembolic complications showed a trend to occur more often in patients with leukapheresis.

Patients with ED_d7_ were in a significant worse performance status (ECOG 3/4 63% vs. 3%), showed higher LDH levels (1568 U/l vs. 926 U/l), more signs of leukostasis (dyspnea 78% vs. 35%; acute renal failure 67% vs. 2%) and a higher degree of coagulation disturbances (prothrombin time 40% vs. 60%; fibrinogen 73 mg/dl vs. 283 mg/dl) compared to patients that survived >7 days (**Table S2**).

### Development of a risk score for Early Death

To address the question whether patients undergoing therapeutic leukapheresis have a higher early mortality because of the more aggressive disease itself and the adverse patient characteristics (higher ECOG performance status, more clinical signs of leukostasis e.g. dyspnea, neurological impairment, disturbed coagulation) or due to the procedure of leukapheresis itself we developed a risk score for ED_d7_.

Therefore, we performed univariate logistic regression analyses with the endpoint ED_d7_ for all available clinical parameters (**Table S3**). We used a significance level of 5%.

Parameters with a univariate significant impact on ED_d7_ included a performance status ≥ ECOG 3/4, dyspnea vs. non dyspnea, prothrombin time, LDH level and creatinine. Age, sex, de novo AML, AML at first diagnosis, FAB type M4/M5, cytogenetic risk, ELN risk, normal karyotype, *NPM1* mutation status, *FLT3-ITD* mutation status, presence of a *FLT3*-TKD, presence of a *MLL-*PTD, WBC, platelet count, haemoglobin level, bone marrow (BM) blasts and peripheral blood (PB) blasts, PTT, fibrinogen, antithrombin III, troponin, lactate levels, D-dimer and neurologic derogation displayed no significant effect on ED_d7_.

In the next step, all univariate significant parameters were tested pairwise in bivariate logistic regression. In pairwise bivariate logistic regression only the prothrombin time, creatinine and the ECOG performance status retained their statistical significance with respect to ED_d7_.

These three bivariate significant parameters were introduced in an inclusion multivariate logistic regression model with a significance level of 5% with and without 999 bootstrap replications, in which lower ECOG3/4, lower prothrombin time and higher creatinine kept their independent adverse prognostic impact on ED_d7_ (**Table 6**). Due to the incomplete documentation of the ECOG performance status, which was available in only 37/52 patients, and the lack of its significance in multivariate logistic regression with backward Wald stepwise selection, the risk score for ED_d7_ was calculated using the regression coefficients Beta from bivariate regression analysis of creatinine and thromboplastin time:
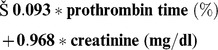



**Table 6:** Multivariate logistic regression for Death within 7 days (Early Death) with inclusion based on 999 bootstraps.

Parameter	Comparison	n	HR	Lower CL	Upper CL	*P*
**Thromboplastin Time (%)**	**+1%**	**52**	**0.918**	**−5.211**	**0.587**	**0.028**
**Creatinine (mg/dl)**	**+1 mg/dl**	**52**	**14.877**	**−19.785**	**108.082**	**0.046**
**ECOG**	**ECOG 3/4vs 0–2**	**37**	**3.316**	**−4.270**	**116.205**	**0.050**

The variables significant in bivariate logistic regression were introduced into a multivariate model with the endpoint ED_d7_.Thromboplastin time, Creatinine and ECOG performance status reached a *P*-value of ≤0.005 which was considered significant.

Abbreviations: HR, Hazard Ratio; CL, Confidence limit, n, number.

The median score was −4.47 (range: −6.84 to 1.68), and the median mortality risk within the first week was 6.7% (range: 0.4% to 99.0%). The risk score showed a highly significant univariate impact when applied into a logistic regression model on ED_d7_, p = 0.002; Odds Ratio (OR): 2.7; 95% CI: 1.5–5.0).

As an internal control, the risk score was introduced with each parameter (age, gender, WBC, platelet count, hemoglobin level, peripheral blood blasts, bone marrow blasts, LDH, troponin, lactate, PTT, fibrinogen, antithrombin III, D-dimer, AML M4/M5, ELN risk group, cytogenetic risk group, cytogenetically normal AML vs. non cytogenetically normal AML, de novo AML, AML at first diagnosis vs. AML at relapse, *NPM1* mutation status, *FLT3-ITD* mutation status, presence of a *FLT3*-TKD, presence of a *MLL-*PTD, clinical parameters such as dyspnea vs. non dyspnea (oxygen requirement), neurologic derogation, shock and acute renal failure) in separate bivariate logistic regression models. No other parameter gained additional significant impact.

Applying the ROC analyses the Area under the curve (AUC) was 0.876 (**Figure S4**). We identified the cutoff −2.81 as optimal cutoff with a sensitivity of 77.8%, a specificity of 90.7%.

Using this cutoff, the score defined two groups a low risk group (lowR): score <−2.81 vs. a high risk group (HiR): score ≥−2.81. A score of −2.81 was equivalent to an ED_d7_ mortality risk of 26.5%. This cutoff separated two groups with different median ED_d7_ mortality risks of 4.9% and 50.1%.

**Figure 4:**
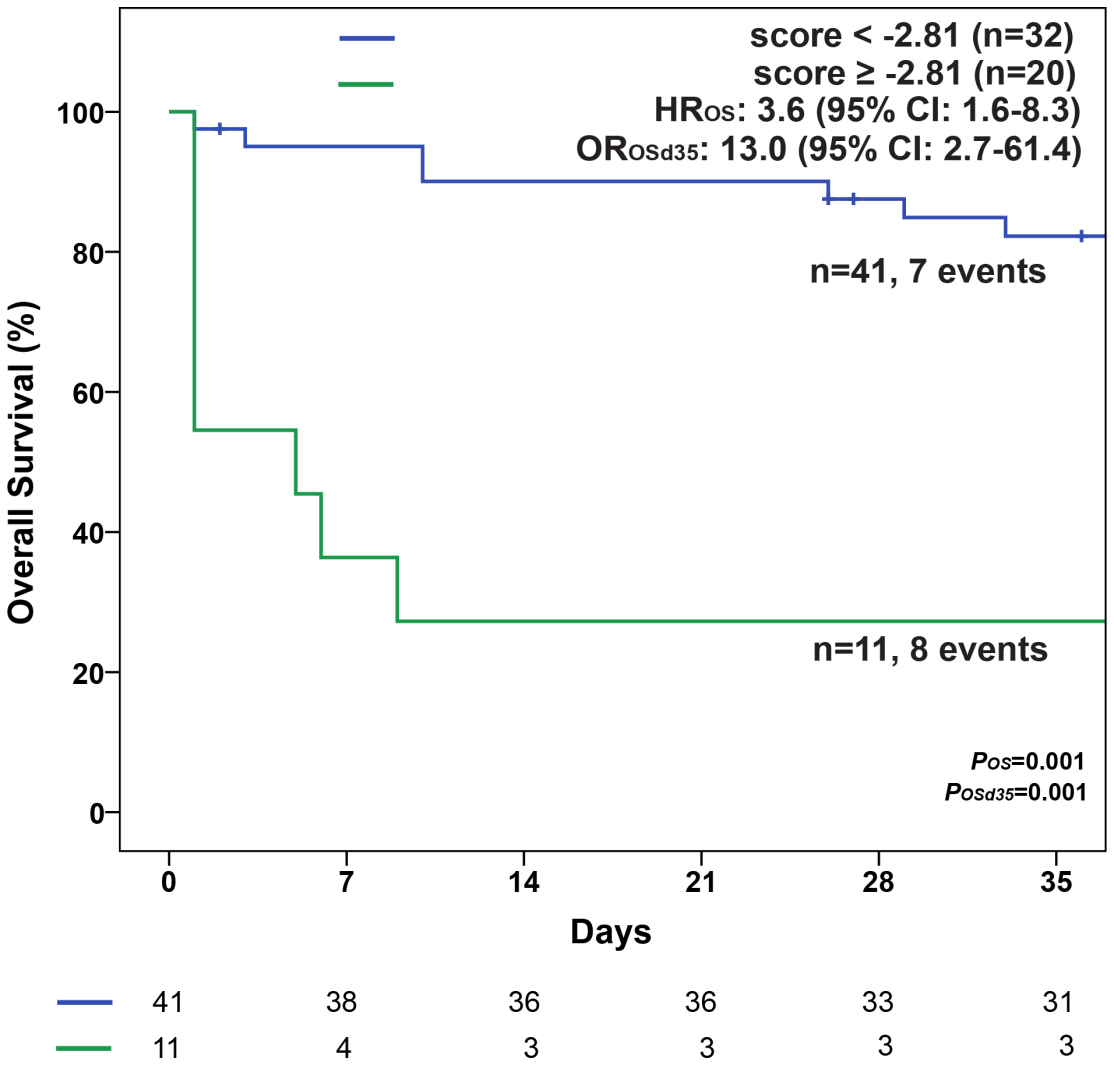
Survival in the first 35 days according to the ED_d7_ Score. Median OS was 8.8 months (95% CI: 5.4–12.3 months) in patients with a LowR score (<−2.81) and 0.2 months (95% CI: 0.03–0.3 months) in patients with a HiR score (≥−2.81). Abbreviations: CI, confidence interval; HR, Hazard Ratio; OR_d35_, Odds ratio for death until (≤) day 35; OS, Overall survival.

Furthermore, these groups showed significant different early mortalities within the first 35 days (p = 0.001) (**Figure 4**) and different overall survival (Median OS: 8.8 months in the LowR vs. 0.2 months in the HiR group; p = 0.001).

### Therapeutic leukapheresis does not significantly influence early mortality

More patients within the HiR group were treated with leukapheresis plus chemotherapy compared to chemotherapy only (64% vs 36%, p = 0.053). About one third of patients that received leukapheresis plus chemotherapy belonged to the HiR group compared to two thirds of patients that were classified as LowR (**Table 7**).

**Table 7:** Distribution of leukapheresis in two risk groups of the ED_d7_ Score.

	ED_d7_ Score		*P*
**Leukapheresis**	**LowR (n = 41)**	**HiR (n = 11)**	
**Yes (n = 20)**	**13/41 (32%)**	**7/11 (64%)**	**0.053**
**No (n = 32)**	**28/41 (68%)**	**4/11 (36%)**	

The group of patients undergoing leukapheresis contained more patients classified as HiR compared to the group of patients that received chemotherapy only.

Abbreviations: HiR, high risk group according to the early death day 7 score; LowR, low risk group according to the early death day 7 score.

In a univariate logistic regression in all patients, patients undergoing leukapheresis showed a trend towards a higher risk for ED_d7_ which was not statistically significant. Further subgroup analyses revealed that neither in patients with a lowR score nor in those with a HiR score, therapeutic leukapheresis had a significant impact on ED_d7_ (**Table 8**).

**Table 8:** Univariate logistic regression for Death within 7 days (ED_d7_).

Group	Comparison	n	OR	Lower CL	Upper CL	*P*
**All patients**	**Leukapheresis vs no leukapheresis**	**52**	**4.143**	**0.901**	**19.049**	**0.068**
**LowR**	**Leukapheresis vs no leukapheresis**	**41**	**2.250**	**0.130**	**39.053**	**0.578**
**HiR**	**Leukapheresis vs no leukapheresis**	**11**	**2.500**	**0.194**	**32.194**	**0.482**

In the entire patient cohort, the effect of leukapheresis was not significant in the context of the early death score.

Abbreviations: CL, confidence limit; HiR, high risk group according to the early death day 7 score; LowR, low risk group according to the early death day 7 score; n, number; OR, Odds Ratio.

To address the independent impact of leukapheresis on early mortality in the setting of other risk factors, we performed a binary logistic regression with the variables “ED_d7_ score” (as a surrogate for all early death markers) and the parameter “leukapheresis”

When the score was introduced into the model either as a continuous or as a dichotomized variable (lowR and the hiR groups), in our patient cohort therapeutic leukapheresis did not have a significant impact on early mortality (**Table 9A and 9B**).

**Table 9:** A: binary logistic regression for Death within 7 days (ED_d7_). B: binary logistic regression for Death within 7 days (ED_d7_).

A: binary logistic regression for Death within 7 days (ED_d7_)						
**Parameter**	**Comparison**	**n**	**OR**	**Lower CL**	**Upper CL**	***P***
**Early Death Score**	**Continuous parameter**	**52**	**2.772**	**1.384**	**5.552**	**0.004**
**Therapeutic leukapheresis**	**yes vs. no**	**52**	**0.853**	**0.105**	**6.920**	**0.882**

B: binary logistic regression for Death within 7 days (ED_d7_)						
**Parameter**	**Comparison**	**n**	**OR**	**Lower CL**	**Upper CL**	***P***
**Early Death Score**	**HiR vs LowR**	**52**	**28.572**	**4.240**	**192.561**	**0.001**
**Therapeutic leukapheresis**	**yes vs. no**	**52**	**2.386**	**0.356**	**15.989**	**0.370**

**9A**: Therapeutic leukapheresis did not impact on the early death rate when the early death score was introduced as a continuous variable into the binary logistic regression. **9B**: Therapeutic leukapheresis might lower the risk of early death in patients belonging to the LowR d7 group. Abbreviations: CL, confidence limit; n, number; OR, Odds Ratio.

## Discussion

Our survival data of hyperleukocytotic AML patients with an early death rate within seven days of 17% confirm the devastating and fatal course of leukostasis in AML as a life threatening clinical condition with multiorgan failure.

Hyperleukocytosis in AML not only increases early mortality, but is also a risk factor for a shorter OS (median 9 months in our cohort) and an overall death and relapse rate of 77%.

We could confirm (**Table 2**) observations of other groups, that hyperleukocytotic AML is often associated with AML FAB types M4 and M5 [22], a high frequency of *FLT3*-ITD and MLL-rearrangements [23].

Median age in our hyperleukocytotic AML cohort was 60 years. This was comparable to the median age of AML onset reported in the literature [24]. In line with data reported by Büchner *et al.* [25] and Oliveira *et al.* [26], older age did not seem to be associated with a higher WBC in our cohort;Leukapheresis is a procedure to rapidly lower WBC recommended by the American Society for Apheresis for AML patients with hyperleukocytosis of >100 G/l [27], [28].

We could also demonstrate that leukapheresis in addition to chemotherapy can reduce WBC significantly faster than chemotherapy alone.

Complications that occurred in the first 7 days after start of treatment included mostly bleeding and thromboembolic events and were not significantly different between the two patient cohorts, although there was a slight trend towards a higher thromboembolic risk in patients that underwent leukapheresis.

We have observed a higher early death rate (ED_d7_) in the leukapheresis group.

Since therapeutic leukapheresis was performed more often in patients with relevant signs of leukostasis-related organ dysfunction (e.g. higher troponin, higher creatinine, lower oxygen saturation) (**Table 3**), we could not distinguish if the worse prognosis of these patients was caused as a complication of therapeutic leukapheresis or occurred due to their underlying impaired clinical condition.

To assess the effect of leukapheresis on ED_d7_ in an independent manner, we have therefore developed a clinical score for the estimation of a hyperleukocytotic AML patient's early mortality based on significant parameters.

Interestingly, multivariate analysis without selection revealed, that only clinical parameters such as serum creatinine, the thromboplastin time and ECOG performance status had an independent influence on ED_d7_, whereas cytogenetic, cytomorphologic and molecular differences in the AML subtypes as well as WBC or platelet count did not have any impact.

Importantly, older age, a known risk factor for a dismal prognosis in AML, did not display a significant impact on ED_d7_ (nor on OS) in these hyperleukocytotic patients and was therefore not included in the calculation of the score.

The three significant parameters creatinine, thromboplastin time and ECOG performance status, reflect the end-organe failure caused by the microangiopathy (creatinine), the coagulopathy (thromboplastin time) and the impairment of the patient condition caused by the aggressive nature of the hyperleukocytotic/leukostatic AML.

The ECOG performance status was reported only in 71% of all patients. Our aim was to define parameters that were significantly associated with early mortality and to investigate the role of leukapheresis in these critical ill patients. We decided not to introduce the ECOG performance status into our risk score, because we did not want to lower the number of assessable patients and because ECOG lost its significance when introduced in multivariate logistic regression with backward Wald selection. Thus, we developed a risk score for ED (ED_d7_ score) on the basis of two parameters, the initial creatinine and thromboplastin time. Nevertheless, we exploratively developed an ED_d7_ score including the three risk parameters creatinine, thrombomplastin time and the ECOG status which showed similar results (data not shown).

Patients grouped in the HiR ED_d7_ category showed significantly more laboratory (troponine, lactate) and clinical (dyspnea, neurologic derogation, renal failure, shock) signs of leukostasis, significant more frequent coagulopathy, a worse ECOG performance status and per definition higher creatinine and lower thromboplastin times (**Table S2**).

The ED_d7_ score was designed to separate patients with a low or high risk with regard to early mortality within one week.

The ED_d7_ score separates two groups with different one month mortality (p = 0.001)) and overall survival (Median OS: 8.8 months in the LowR vs. 0.2 months in the HiR group; p = 0.001). In the HiR group and LowR groups, 63.6% and 4.9% of the patients died in the first week. Despite the better early survival in LowR patients compared to HiR patients, mortality in these cohorts within the first 24 months when censoring for allogeneic transplantation was comparable, 85.1% and 86.4% respectively. These observations reflect the dismal prognosis in hyperleukocytotic AML patients depending on acute complications and multiorgan failure within the first week after diagnosis as well as the unfavorable biology of hyperleukocytotic AML itself associated with a higher relapse rate and adverse outcome.

In our patient cohort, we could not detect any evidence that leukapheresis did have an influence on early mortality. Leukapheresis did not affect the risk of ED_d7_ in all patients, nor in the lowR neither in the HiR patients defined by our score. Although, in our patient cohort leukapheresis showed a trend towards a higher ED_d7_ rate.

Nevertheless, our results are limited by the retrospective nature of investigation and the small patient numbers. Furthermore, the establishment of a risk score of ED_d7_ in 52 patients with a large number of tested variables (n = 29) might bear the risk of identification of false-positive results.

Therefore, larger and prospective, preferably multicenter, trials are needed to confirm the score which might be a useful tool to address the important clinical question if leukapheresis should be performed in hyperleukocytotic AML patients.

## Supporting Information

Figure S1 **WBC before and after 1 leukapheresis.** Leukapheresis (LA) significantly reduces WBC (p<0.001, paired sample t-test). A box is limited by the 25th and the 75^th^ quantile, the median is illustrated by the dashed line within the box. Abbreviations: LA, leukapheresis; WBC, white blood cell count.
(TIF)

Figure S2 **Event-free survival (EFS) in patients with WBC≥100 G/l.** (**A**) in all patients (**B**) in patients who received either chemotherapy only or chemotherapy combined with leukapheresis. Median EFS was 3.3 months (95% CI: 2.0–4.7 months) in all patients, 3.3 months (95% CI: 2.2–4.4 months) in patients receiving chemotherapy only and 3.7 months (95% CI: 0.7–6.6 months) in patients with the combination of chemotherapy and leukapheresis. Abbreviations: CI, Confidence Interval; HR, Hazard Ratio; n, number.
(TIF)

Figure S3 **Cumulative Incidence of Relapse (CIR) in patients with WBC≥100 G/l in complete remission.** (**A**) in all patients (**B**) in patients who received either chemotherapy only or chemotherapy combined with leukapheresis. Median time from CR to relapse was 6.2 months (95% CI: 2.9–9.6 months) in all patients, 8.0 months (95% CI: 2.8–13.3 months) in patients receiving chemotherapy only and 3.2 months (95% CI: 0.1–6.9 months) in patients with the combination of chemotherapy and leukapheresis. Abbreviations: CI, Confidence Interval; CR, Complete Remission; HR, Hazard Ratio; n, number.
(TIF)

Figure S4 **Receiver operating characteristic (ROC) curve for the Early Death Score.** The Early Death d7 score has a high predictive value. Abbreviation: AUC, Area under the curve; CI, Confidence Interval.
(TIF)

Table S1 **Therapeutic regimen in patients with WBC≥100 G/l.** Overview of therapeutic regimen in patients with hyperleukocytosis. Abbreviations: CR =  complete remission; FLAG-IDA, fludarabine (30 mg/m^2^/d, days 1–4, intravenously), cytarabine (2 g/m^2^/d, days 1–4, intravenously), idarubicin (12 mg/m^2^/d, days 2–4, intravenously), filgrastim (400 µg/m^2^/d, day 0 up to neutrophil count >1×10^9^/l, subcutaneously); FLAMSA, fludarabine (30 mg/m^2^/d, days 1–4, intravenously), amsacrine (100 mg/m^2^/d, days 1–4 intravenously), cytarabine (2 g/m^2^/d, days 1–4, intravenously); FS-HAI, fludarabine (15 mg/m^2/^/bid 4 h before each cytarabine, days 1–2 and 8–9, intravenously) sequential high dose cytarabine (1 g/m^2^/bid, days 1–2 and 8–9, intravenously), idarubicin (10 mg/m^2^/d, days 3–4 and 10–11, intravenously); HAM, high dose cytarabine (3 g/m^2^/bid in patients <60 years or 1 g/m^2^/bid in patients ≥60 years, days 1–3, intravenously) mitoxantrone (10 mg/m^2^/d, days 3–5, intravenously); n = number; sHAM, sequential high dose cytarabine (3 g/m^2^/bid in patients <60 years or 1 g/m^2^/bid in patients ≥60 years, days 1–2, 8–9, intravenously), mitoxantrone (10 mg/m^2^/d, days 3–4, 10–11, intravenously); TAD, thioguanine (100 mg/m^2^/bid, days 3–9, orally), cytarabine (100 mg/m^2^/d, days continuous infusion, days 1–2 and 100 mg/m^2^/bid, days 3–8, intravenously) daunorubicin (60 mg/m^2^/d, days 3–5, intravenously), WBC, white blood count; 7+3, cytarabine (100 mg/m^2^/continuous infusion, days 1–7, intravenously), anthracycline (daunorubicin: 60 mg/m^2^/d, days 3–5, intravenously).
(DOCX)

Table S2 **Comparisons of patients with and without early death within 7 days (ED_d7_).** Patients who died within the first seven days after diagnosis showed a worse ECOG, higher LDH levels, a more severe disturbance of coagulation (lower prothrombin time and lower fibrinogen levels) and more clinical signs of leukostasis compared to patients that survived after day 7. Abbreviations: BM blasts; bone marrow blasts; CPAP, continuous positive airway pressure; EOCG, Eastern Cooperative Group; FAB, French-American-British classification of AML; *FLT*3-ITD, internal tandem duplication of the *FLT*3 gene; *FLT*3-TKD, point mutation at D835 in the *FLT*3-tyrosine kinase domain of the *FLT*3 gene; HD, hemodialysis; HF, hemofiltration; ITN, intubaton; *MLL*-PTD, partial tandem duplication of the *MLL* gene; n, number; *NPM*1, nucleophosmin1; PB blasts, blasts in the peripheral blood; PTT, partial thromboplastin time; WBC, white blood count.
(DOCX)

Table S3 **Unviarate logistic regression for early death within 7 days (ED_d7_).** Overview of significant parameters for death within the first seven days in a univariate logistic regression. Abbreviations: BM blasts; bone marrow blasts; CL; confidence limit; CPAP, continuous positive airway pressure; ELN, European Leukemia Net classification of AML; EOCG, Eastern Cooperative Group; FAB, French-American-British classification of AML; *FLT*3-ITD, internal tandem duplication of the *FLT*3 gene; *FLT*3-TKD, point mutation at D835 in the *FLT*3-tyrosine kinase domain of the *FLT*3 gene; HD, hemodialysis; HF, hemofiltration; ITN, intubaton; LDH, lactase dehydrogenase level; *MLL*-PTD, partial tandem duplication of the *MLL* gene; NA, not applicable, n.s., not significant; n, number; *NPM*1, nucleophosmin1; OR, Odds Ratio; PB blasts; blasts in the peripheral blood; PTT, partial thromboplastin time; WBC, white blood count.
(DOCX)

Checklist S1 **TREND Checklist.**
(PDF)
