# Supplementary figures and images for: The Role of Therapeutic Leukapheresis in Hyperleukocytotic AML
Source: PLoS One. 2014 Apr 14;9(4):e95062. doi: 10.1371/journal.pone.0095062 (PMC3986260; doi:10.1371/journal.pone.0095062)

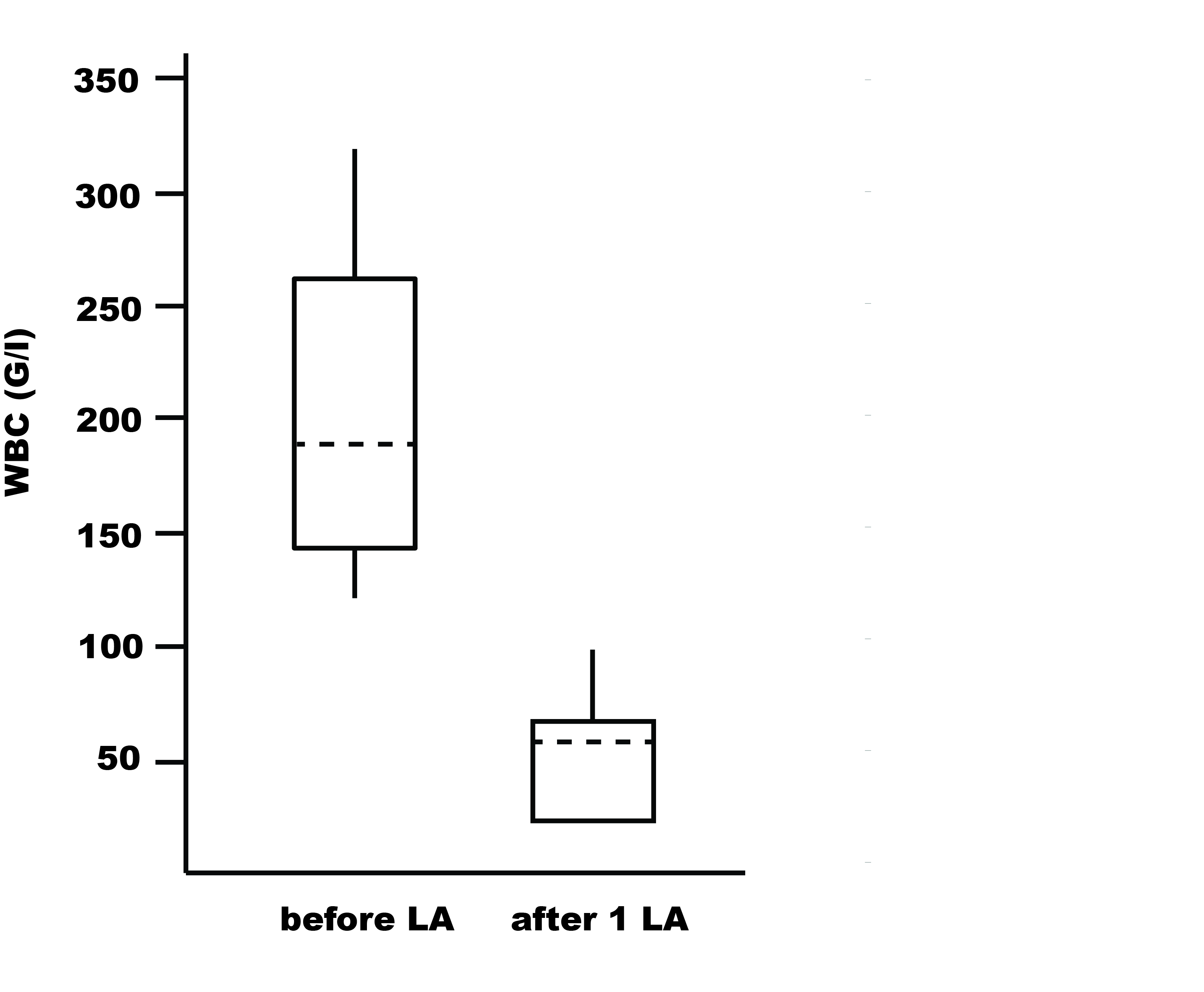

Supplement: Figure S1 — WBC before and after 1 leukapheresis. Leukapheresis (LA) significantly reduces WBC (p<0.001, paired sample t-test). A box is limited by the 25th and the 75th quantile, the median is illustrated by the dashed line within the box. Abbreviations: LA, leukapheresis; WBC, white blood cell count. (TIF) [file pone.0095062.s001.tif]

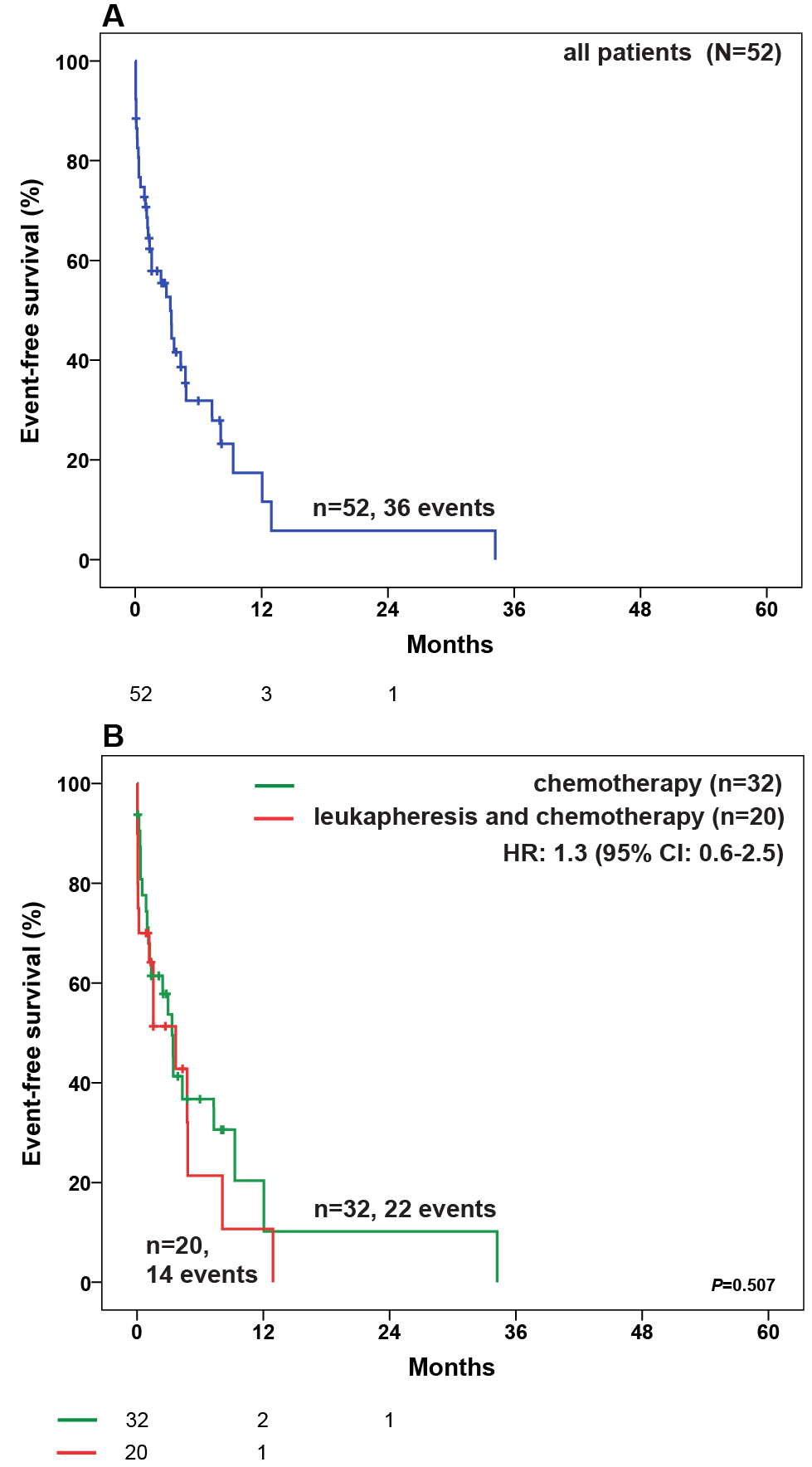

Supplement: Figure S2 — Event-free survival (EFS) in patients with WBC≥100 G/l. (A) in all patients (B) in patients who received either chemotherapy only or chemotherapy combined with leukapheresis. Median EFS was 3.3 months (95% CI: 2.0–4.7 months) in all patients, 3.3 months (95% CI: 2.2–4.4 months) in patients receiving chemotherapy only and 3.7 months (95% CI: 0.7–6.6 months) in patients with the combination of chemotherapy and leukapheresis. Abbreviations: CI, Confidence Interval; HR, Hazard Ratio; n, number. (TIF) [file pone.0095062.s002.tif]

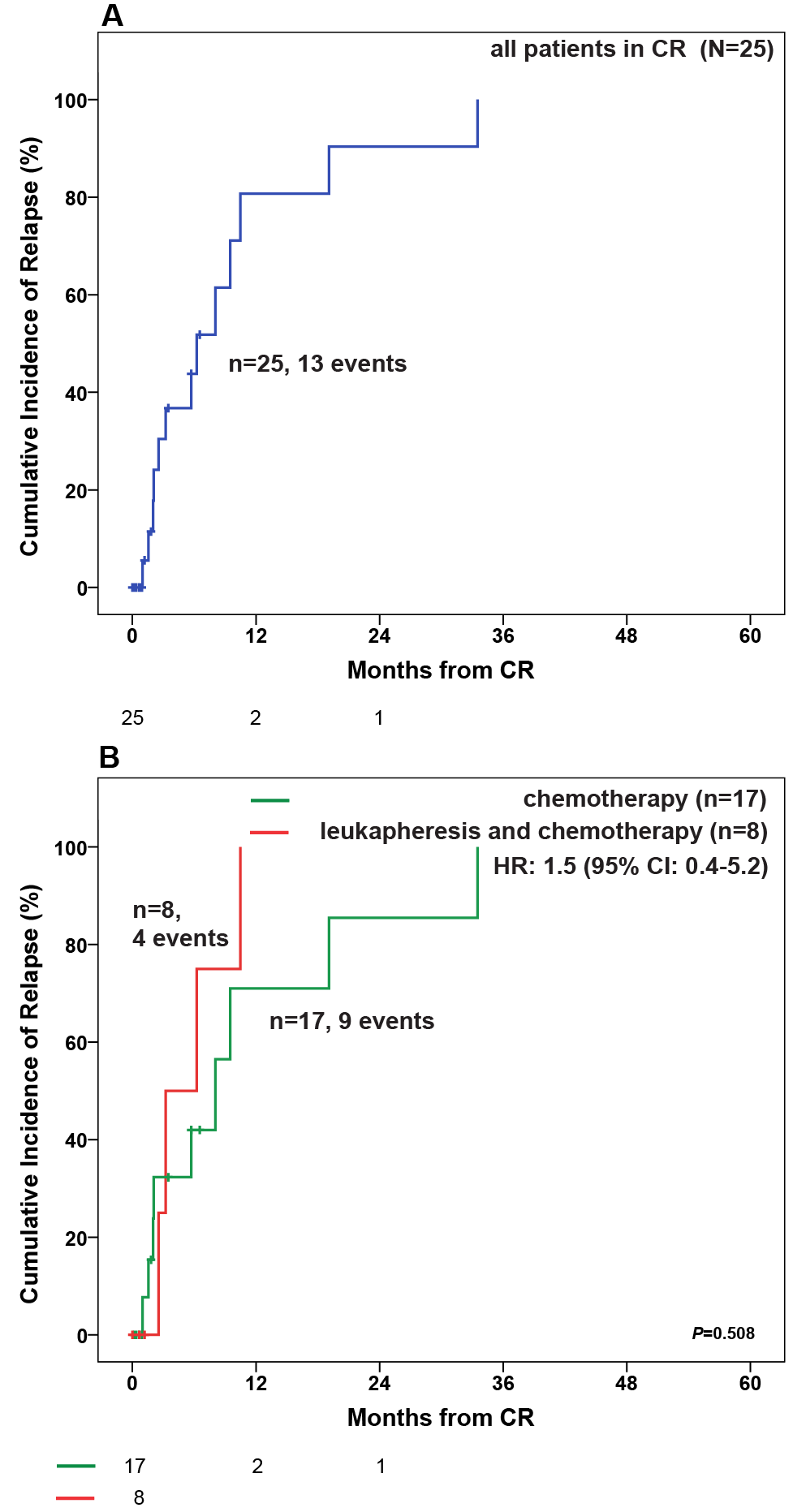

Supplement: Figure S3 — Cumulative Incidence of Relapse (CIR) in patients with WBC≥100 G/l in complete remission. (A) in all patients (B) in patients who received either chemotherapy only or chemotherapy combined with leukapheresis. Median time from CR to relapse was 6.2 months (95% CI: 2.9–9.6 months) in all patients, 8.0 months (95% CI: 2.8–13.3 months) in patients receiving chemotherapy only and 3.2 months (95% CI: 0.1–6.9 months) in patients with the combination of chemotherapy and leukapheresis. Abbreviations: CI, Confidence Interval; CR, Complete Remission; HR, Hazard Ratio; n, number. (TIF) [file pone.0095062.s003.tif]

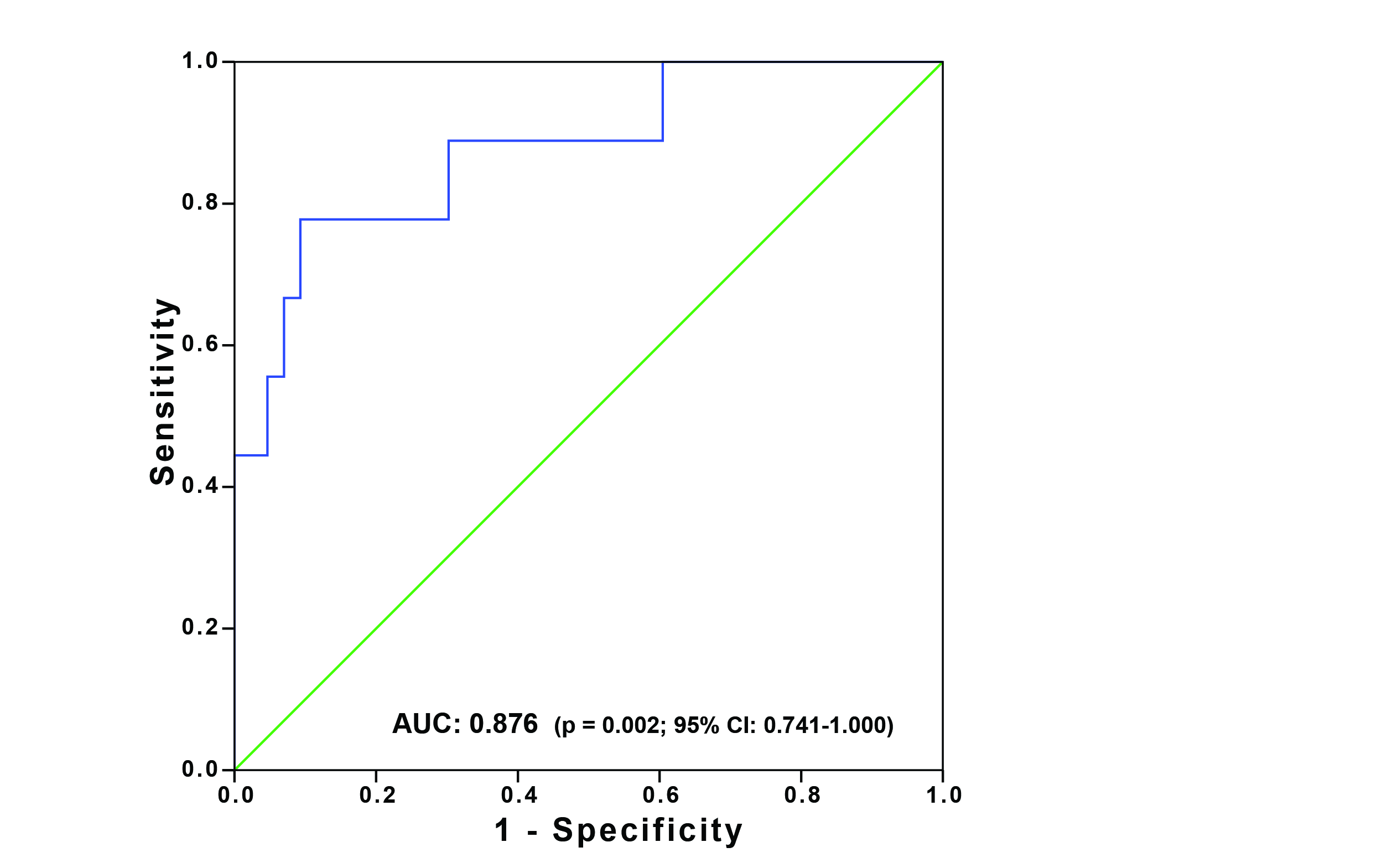

Supplement: Figure S4 — Receiver operating characteristic (ROC) curve for the Early Death Score. The Early Death d7 score has a high predictive value. Abbreviation: AUC, Area under the curve; CI, Confidence Interval. (TIF) [file pone.0095062.s004.tif]
