# Supplementary material for: The Role of Therapeutic Leukapheresis in Hyperleukocytotic AML
Source: PLoS One. 2014 Apr 14;9(4):e95062. doi: 10.1371/journal.pone.0095062 (PMC3986260; doi:10.1371/journal.pone.0095062)
Supplement: Table S1 — Therapeutic regimen in patients with WBC≥100 G/l. Overview of therapeutic regimen in patients with hyperleukocytosis. Abbreviations: CR = complete remission; FLAG-IDA, fludarabine (30 mg/m2/d, days 1–4, intravenously), cytarabine (2 g/m2/d, days 1–4, intravenously), idarubicin (12 mg/m2/d, days 2–4, intravenously), filgrastim (400 µg/m2/d, day 0 up to neutrophil count >1×10^9^/l, subcutaneously); FLAMSA, fludarabine (30 mg/m2/d, days 1–4, intravenously), amsacrine (100 mg/m2/d, days 1–4 intravenously), cytarabine (2 g/m2/d, days 1–4, intravenously); FS-HAI, fludarabine (15 mg/m2//bid 4 h before each cytarabine, days 1–2 and 8–9, intravenously) sequential high dose cytarabine (1 g/m2/bid, days 1–2 and 8–9, intravenously), idarubicin (10 mg/m2/d, days 3–4 and 10–11, intravenously); HAM, high dose cytarabine (3 g/m2/bid in patients <60 years or 1 g/m2/bid in patients ≥60 years, days 1–3, intravenously) mitoxantrone (10 mg/m2/d, days 3–5, intravenously); n = number; sHAM, sequential high dose cytarabine (3 g/m2/bid in patients <60 years or 1 g/m2/bid in patients ≥60 years, days 1–2, 8–9, intravenously), mitoxantrone (10 mg/m2/d, days 3–4, 10–11, intravenously); TAD, thioguanine (100 mg/m2/bid, days 3–9, orally), cytarabine (100 mg/m2/d, days continuous infusion, days 1–2 and 100 mg/m2/bid, days 3–8, intravenously) daunorubicin (60 mg/m2/d, days 3–5, intravenously), WBC, white blood count; 7+3, cytarabine (100 mg/m2/continuous infusion, days 1–7, intravenously), anthracycline (daunorubicin: 60 mg/m2/d, days 3–5, intravenously). (DOCX) [file pone.0095062.s005.docx]

| **TABLE S1: Therapeutic regimen in patients with WBC ≥ 100 G/l** | | | | |
| --- | --- | --- | --- | --- |
|  |  | **n=52** | |  |
| **therapy** | **n** |  | | **%** |
| **Leukapheresis** | **20** |  | **39** | |
| **Induction cycles** |  |  |  | |
| **none** | **12** |  | **23** | |
| **single induction course at first diagnosis** | **13** |  | **25** | |
| **7+3** | **4** |  | **31** | |
| **TAD** | **5** |  | **38** | |
| **HAM** | **4** |  | **31** | |
| **single induction course at relapse** | **4** |  | **8** | |
| **FLAG-IDA** | **1** |  | **25** | |
| **FS-HAI** | **1** |  | **25** | |
| **7+3** | **2** |  | **50** | |
| **double induction course** | **23** |  | **44** | |
| **7+3 - HAM** | **4** |  | **18** | |
| **TAD - TAD** | **1** |  | **4** | |
| **TAD - HAM** | **8** |  | **35** | |
| **HAM - HAM** | **1** |  | **4** | |
| **sHAM** | **8** |  | **35** | |
| **7+3 - FLAMSA** | **1** |  | **4** | |
| **Allogeneic transplantation** | **17** |  | **33** | |
| **in 1. CR** | **10** |  | **59** | |
| **relapse** | **5** |  | **29** | |
| **primary refractory** | **2** |  | **12** | |
