# Supplementary material for: The Role of Therapeutic Leukapheresis in Hyperleukocytotic AML
Source: PLoS One. 2014 Apr 14;9(4):e95062. doi: 10.1371/journal.pone.0095062 (PMC3986260; doi:10.1371/journal.pone.0095062)
Supplement: Table S2 — Comparisons of patients with and without early death within 7 days (EDd7). Patients who died within the first seven days after diagnosis showed a worse ECOG, higher LDH levels, a more severe disturbance of coagulation (lower prothrombin time and lower fibrinogen levels) and more clinical signs of leukostasis compared to patients that survived after day 7. Abbreviations: BM blasts; bone marrow blasts; CPAP, continuous positive airway pressure; EOCG, Eastern Cooperative Group; FAB, French-American-British classification of AML; FLT3-ITD, internal tandem duplication of the FLT3 gene; FLT3-TKD, point mutation at D835 in the FLT3-tyrosine kinase domain of the FLT3 gene; HD, hemodialysis; HF, hemofiltration; ITN, intubaton; MLL-PTD, partial tandem duplication of the MLL gene; n, number; NPM1, nucleophosmin1; PB blasts, blasts in the peripheral blood; PTT, partial thromboplastin time; WBC, white blood count. (DOCX) [file pone.0095062.s006.docx]

| **TABLE S2: Comparisons of patients with and without early death within 7 days (ED _d7_)** | | | | | | | | |
| --- | --- | --- | --- | --- | --- | --- | --- | --- |
| **Characteristic** |  | | **Early death _d7_ (n=11)** |  |  | **No Early death _d7_ (n=41)** |  | ***P*** |
|  | **n** |  | | **%** | **n** |  | **%** |  |
| **Age (years)** |  |  | |  |  |  |  | **0.358** |
| **median** |  | **51** | |  |  | **62** |  |  |
| **range** |  | **21-78** | |  |  | **31-79** |  |  |
| **Female sex** | **4/11** |  | | **36** | **26/41** |  | **63** | **0.107** |
| **ECOG 3/4 n=37** | **7/11** |  | | **64** | **3/37** |  | **8** | **<0.001** |
| **De novo AML** | **9/11** |  | | **82** | **33/41** |  | **81** | **0.921** |
| **First diagnosis** | **10/11** |  | | **91** | **33/41** |  | **81** | **0.417** |
| **WBC (G/l)** |  |  | |  |  |  |  | **0.712** |
| **median** |  | **189** | |  |  | **153** |  |  |
| **range** |  | **100-322** | |  |  | **100-320** |  |  |
| **Platelets (G/l)** |  |  | |  |  |  |  | **0.646** |
| **median** |  | **47** | |  |  | **45** |  |  |
| **range** |  | **25-126** | |  |  | **10-149** |  |  |
| **Hemoglobin level (g/dl)** |  |  | |  |  |  |  | **0.737** |
| **median** |  | **9.6** | |  |  | **9.5** |  |  |
| **range** |  | **5.0-12.9** | |  |  | **6.0-14.9** |  |  |
| **LDH level (U/l) n=46** |  |  | |  |  |  |  | **0.184** |
| **median** |  | **1140** | |  |  | **962** |  |  |
| **range** |  | **575-7387** | |  |  | **444-5411** |  |  |
| **BM blasts (%) n=40** |  |  | |  |  |  |  | **0.648** |
| **median** |  | **87** | |  |  | **87** |  |  |
| **range** |  | **76-88** | |  |  | **46-99** |  |  |
| **PB blasts (%) n=47** |  |  | |  |  |  |  | **0.330** |
| **median** |  | **82** | |  |  | **87** |  |  |
| **range** |  | **39-97** | |  |  | **14-99** |  |  |
| **Creatinine (mg/dl)** |  |  | |  |  |  |  | **0.004** |
| **median** |  | **1.9** | |  |  | **1.1** |  |  |
| **range** |  | **0.9-3.5** | |  |  | **0.6-2.6** |  |  |
| **Troponin (ng/ml) n=23** |  |  | |  |  |  |  | **0.052** |
| **median** |  | **0.19** | |  |  | **<0.05** |  |  |
| **range** |  | **0.06-3.99** | |  |  | **<0.05-10.90** |  |  |
| **Troponin (>0.05 ng/ml) n=23** | **6/6** |  | | **100** | **9/17** |  | **53** | **0.037** |
| **Lactate (mmol/l) n=31** |  |  | |  |  |  |  |  |
| **median** |  | **3.2** | |  |  | **1.1** |  | **0.007** |
| **range** |  | **0.8-36.2** | |  |  | **0.3-3.6** |  |  |
| **Lactate (> 2.4 mmol/l) n=31** | **6/10** |  | | **60** | **1/21** |  | **5** | **0.001** |
| **PTT (sec) n=51** |  |  | |  |  |  |  | **0.011** |
| **median** |  | **41** | |  |  | **34** |  |  |
| **range** |  | **27-141** | |  |  | **20-59** |  |  |
| **Prothrombin time (%)** |  |  | |  |  |  |  | **<0.001** |
| **median** |  | **39** | |  |  | **65** |  |  |
| **range** |  | **6-65** | |  |  | **46-85** |  |  |
| **Fibrinogen (mg/dl) n=35** |  |  | |  |  |  |  | **0.009** |
| **median** |  | **98** | |  |  | **307** |  |  |
| **range** |  | **52-364** | |  |  | **71-577** |  |  |
| **Antithrombin (%) n=31** |  |  | |  |  |  |  | **0.004** |
| **median** |  | **61** | |  |  | **92** |  |  |
| **range** |  | **11-102** | |  |  | **61-129** |  |  |
| **D-dimer (µg/ml) n=15** |  |  | |  |  |  |  | **0.679** |
| **median** |  | **26.9** | |  |  | **22.4** |  |  |
| **range** |  | **2.8-40.0** | |  |  | **0.0-40.0** |  |  |
| **FAB M4/M5 n=51** | **6/11** |  | | **55** | **17/40** |  | **43** | **0.477** |
| **Cytogenetic risk (Byrd) n=49** |  |  | |  |  |  |  | **0.116** |
| **Cytogenetic risk favorable** | **1/8** |  | | **12** | **1/41** |  | **3** |  |
| **Cytogenetic risk intermediate** | **5/8** |  | | **63** | **37/41** |  | **90** |  |
| **Cytogenetic risk adverse** | **2/8** |  | | **25** | **3/41** |  | **7** |  |
| **ELN risk (Döhner) n=45** |  |  | |  |  |  |  | **0.966** |
| **ELN favorable** | **2/8** |  | | **25** | **8/37** |  | **22** |  |
| **ELN intermediate I** | **3/8** |  | | **37** | **17/37** |  | **46** |  |
| **ELN intermediate II** | **2/8** |  | | **25** | **7/37** |  | **19** |  |
| **ELN adverse** | **1/8** |  | | **12** | **5/37** |  | **13** |  |
| **Karyotype n=50** |  |  | |  |  |  |  |  |
| **Cytogenetically normal** | **3/8** |  | | **38** | **27/41** |  | **66** | **0.132** |
| ***NPM*1*+* n=41** | **2/7** |  | | **25** | **17/34** |  | **47** | **0.301** |
| ***FLT*3-ITD+ n=46** | **2/8** |  | | **33** | **18/38** |  | **45** | **0.246** |
| ***FLT*3-TKD+ n=38** | **0/3** |  | | **0** | **3/35** |  | **9** | **0.597** |
| ***MLL*-PTD+ n=42** | **0/6** |  | | **0** | **2/36** |  | **6** | **0.554** |
| **Clinical signs of leukostasis** |  |  | |  |  |  |  |  |
| **Dyspnea n=46** | **8/10** |  | | **80** | **12/36** |  | **33** | **0.008** |
| **Oxygen requirement n=46** |  |  | |  |  |  |  | **<0.001** |
| **none** | **2/10** |  | | **20** | **24/36** |  | **67** |  |
| **nasal prongs (≤ 4l O2/min)** | **1/10** |  | | **10** | **9/36** |  | **25** |  |
| **oxygen mask (> 5l O2/min)** | **4/10** |  | | **40** | **3/36** |  | **8** |  |
| **CPAP/ITN** | **3/10** |  | | **30** | **0/36** |  | **0** |  |
| **Neurologic derogation n=50** | **3/11** |  | | **27** | **1/29** |  | **3** | **0.008** |
| **Acute renal failure / HD/HF/Creatinine > 1.5 mg/dl** | **8/11** |  | | **73** | **5/41** |  | **12** | **<0.001** |
| **Any cause of shock** | **4/11** |  | | **36** | **1/41** |  | **2** | **0.001** |
