# Supplementary material for: The Role of Therapeutic Leukapheresis in Hyperleukocytotic AML
Source: PLoS One. 2014 Apr 14;9(4):e95062. doi: 10.1371/journal.pone.0095062 (PMC3986260; doi:10.1371/journal.pone.0095062)
Supplement: Table S3 — Unviarate logistic regression for early death within 7 days (EDd7). Overview of significant parameters for death within the first seven days in a univariate logistic regression. Abbreviations: BM blasts; bone marrow blasts; CL; confidence limit; CPAP, continuous positive airway pressure; ELN, European Leukemia Net classification of AML; EOCG, Eastern Cooperative Group; FAB, French-American-British classification of AML; FLT3-ITD, internal tandem duplication of the FLT3 gene; FLT3-TKD, point mutation at D835 in the FLT3-tyrosine kinase domain of the FLT3 gene; HD, hemodialysis; HF, hemofiltration; ITN, intubaton; LDH, lactase dehydrogenase level; MLL-PTD, partial tandem duplication of the MLL gene; NA, not applicable, n.s., not significant; n, number; NPM1, nucleophosmin1; OR, Odds Ratio; PB blasts; blasts in the peripheral blood; PTT, partial thromboplastin time; WBC, white blood count. (DOCX) [file pone.0095062.s007.docx]

| **Table S3: Unviarate logistic regression for early death within 7 days (ED _d7_)** | | | | | | |
| --- | --- | --- | --- | --- | --- | --- |
| **Parameter** | **Comparison** | **n** | **OR** | **Lower CL** | **Upper CL** | ***P*** |
| **Age (years)** | **+ 10 years** | **52** | **1.304** | **0.747** | **2.275** | **0.351** |
| **Sex** | **Female vs. male** | **52** | **1.583** | **0.349** | **7.174** | **0.551** |
| **ECOG performance status** | **3-4 vs.0-2** | **37** | **46.667** | **4.007** | **543.545** | **0.002** |
| **Origin of AML** | **de novo vs. non-de novo** | **52** | **0.800** | **0.139** | **4.600** | **0.803** |
| **Timepoint of hyperleukocytosis** | **first diagnosis vs. relapse** | **52** | **1.829** | **0.199** | **16.883** | **0.594** |
| **WBC (G/l)** | **10 fold** | **52** | **0.436** | **0.005** | **40.551** | **0.720** |
| **Platelets (G/l)** | **10 fold** | **52** | **3.014** | **0.136** | **66.808** | **0.485** |
| **Hemoglobin level (g/dl)** | **+ 1g/l** | **52** | **0.774** | **0.507** | **1.183** | **0.236** |
| **LDH level (U/l)** | **10 fold** | **49** | **13.114** | **1.180** | **145.747** | **0.036** |
| **BM blasts (%)** | **+ 1%** | **40** | **1.123** | **0.921** | **1.369** | **0.253** |
| **PB blasts (%)** | **+ 1%** | **47** | **0.989** | **0.950** | **1.030** | **0.592** |
| **Creatinine (mg/dl)** | **+ 1mg/dl** | **52** | **3.538** | **1.209** | **10.354** | **0.021** |
| **Troponin (ng/ml)** | **+1ng/ml** | **23** | **0.733** | **0.214** | **2.510** | **0.620** |
| **Lactate (mmol/l)** | **+1mmol/l** | **31** | **1.301** | **0.880** | **1.923** | **0.187** |
| **PTT (sec)** | **+1 sec** | **51** | **1.047** | **0.998** | **1.097** | **0.061** |
| **Prothrombin time (%)** | **+1%** | **52** | **0.906** | **0.850** | **0.966** | **0.002** |
| **Fibrinogen (mg/dl)** | **+1mg/dl** | **35** | **0.990** | **0.980** | **1.000** | **0.057** |
| **Antithrombin (%)** | **+1%** | **31** | **0.956** | **0.911** | **0.990** | **0.062** |
| **D-Dimer (µg/ml)** | **+1µg/ml** | **15** | **0.999** | **0.931** | **1.073** | **0.988** |
| **FAB** | **M4/M5 vs. non-M4/M5** | **51** | **1.263** | **0.279** | **5.723** | **0.762** |
| **Cytogenetic risk**[**^11^**](#_ENREF_11) | **Favorable vs. intermediate vs. adverse** | **49** |  |  |  | **0.215** |
| **ELN risk**[**^20^**](#_ENREF_20) | **ELN favorable vs. intermediate vs. intermediate II vs. adverse** | **45** |  |  |  | **0.589** |
| **Normal Karyotype** | **Cytogenetically normal vs. non-cytogenetically normal** | **46** | **0.593** | **0.107** | **3.295** | **0.550** |
| ***NPM*1** | **pos. vs. neg.** | **41** | **1.176** | **0.149** | **9.266** | **0.887** |
| ***FLT*3-ITD** | **pos. vs. neg.** | **46** | **0.611** | **0.100** | **3.727** | **0.593** |
| ***FLT*3-TKD** | **pos. vs. neg.** | **38** | **NA** | **NA** | **NA** | **0.999** |
| ***MLL*-PTD** | **pos. vs. neg.** | **42** | **NA** | **NA** | **NA** | **0.999** |
| **Oxygen requirement** | **pos. vs. neg.** | **46** | **6.462** | **1.168** | **35.736** | **0.032** |
| **Neurologic derogation** | **pos. vs. neg.** | **50** | **5.571** | **0.670** | **46.354** | **0.112** |
